# Supplementary figures and images for: Comparing the Effects of Differential and Visuo-Motor Training on Functional Performance, Biomechanical, and Psychological Factors in Athletes after ACL Reconstruction: A Randomized Controlled Trial
Source: J Clin Med. 2023 Apr 13;12(8):2845. doi: 10.3390/jcm12082845 (PMC10142379; doi:10.3390/jcm12082845)

## File S1. CONSORT 2010 Flow Diagram

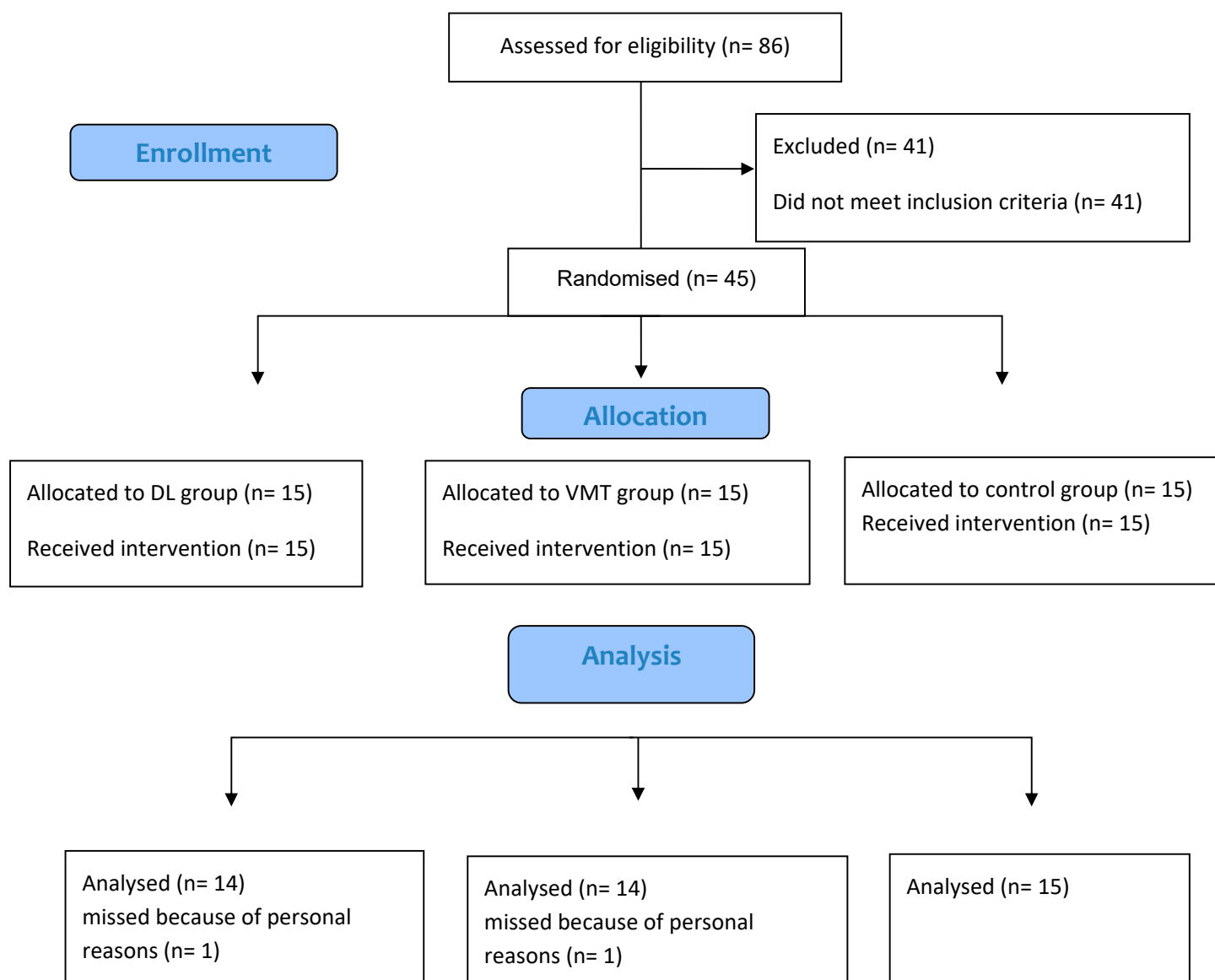

Supplement: Supplementary file 1 [file jcm-12-02845-s001.zip › jcm-2305533-supplementary.pdf]
